# Phylogeography by diffusion on a sphere: whole world phylogeography

**DOI:** 10.7717/peerj.2406

**Published:** 2016-09-06

**Authors:** Remco Bouckaert

**Affiliations:** 1 Centre of Computational Evolution, University of Auckland, Auckland, New Zealand; 2 Max Planck Institute for the Science of Human History, Jena, Germany

**Keywords:** Phylogegraphy, Bayesian analysis, Statistical phylogenetics, Diffusion, BEAST

## Abstract

**Background:**

Techniques for reconstructing geographical history along a phylogeny can answer many questions of interest about the geographical origins of species. Bayesian models based on the assumption that taxa move through a diffusion process have found many applications. However, these methods rely on diffusion processes on a plane, and do not take the spherical nature of our planet in account. Performing an analysis that covers the whole world thus does not take in account the distortions caused by projections like the Mercator projection.

**Results:**

In this paper, we introduce a Bayesian phylogeographical method based on diffusion on a sphere. When the area where taxa are sampled from is small, a sphere can be approximated by a plane and the model results in the same inferences as with models using diffusion on a plane. For taxa sampled from the whole world, we obtain substantial differences. We present an efficient algorithm for performing inference in a Markov Chain Monte Carlo (MCMC) algorithm, and show applications to small and large samples areas. We compare results between planar and spherical diffusion in a simulation study and apply the method by inferring the origin of Hepatitis B based on sequences sampled from Eurasia and Africa.

**Conclusions:**

We describe a framework for performing phylogeographical inference, which is suitable when the distortion introduced by map projections is large, but works well on a smaller scale as well. The framework allows sampling tips from regions, which is useful when the exact sample location is unknown, and placing prior information on locations of clades in the tree. The method is implemented in the GEO_SPHERE package in BEAST 2, which is open source licensed under LGPL and allows joint tree and geography inference under a wide range of models.

## Introduction

Reconstructions of the distribution of species through space and time has been fascinating through the ages (Gee et al., 2006). The abundance of DNA data these days makes it possible to reconstruct species distributions through time by estimating a phylogeny representing the historical relations between species (Drummond et al., 2006). Phylogeography extends a tree with geographical locations, thus allowing inference of species histories through space and time. Random walk models offer a powerful method for describing migration, and can be described mathematically using well understood diffusion processes (Bouckaert et al., 2012; Lemey et al., 2010; Pybus et al., 2012).

A common situation is where we have a tree where the leaf nodes have known point locations and we want to infer the locations of internal nodes, in particular the root location which represents the origin of all taxa associated with leaf nodes in the tree. The tree is typically informed by sequence information associated with the leaf nodes. A number of Bayesian phylogeographical methods have been developed in recent years that make it convenient to combine a phylogenetic analysis with geographical inference. This allows us to answer questions about geographical origins of, for example, viral outbreaks like Ebola (Gire et al., 2014), and HIV (Faria et al., 2014), the Indo-European language family (Bouckaert et al., 2012) as well as many other species (see Drummond & Bouckaert (2015) for more). Some of these methods rely on the taxon locations to be discretised into demes and are based on coalescent theory (Vaughan et al., 2014; De Maio et al., 2015) or ancestral reconstruction (Lemey et al., 2009). Other methods are based on diffusion processes (Bouckaert et al., 2012; Lemey et al., 2010; Pybus et al., 2012).

**Figure 1:**
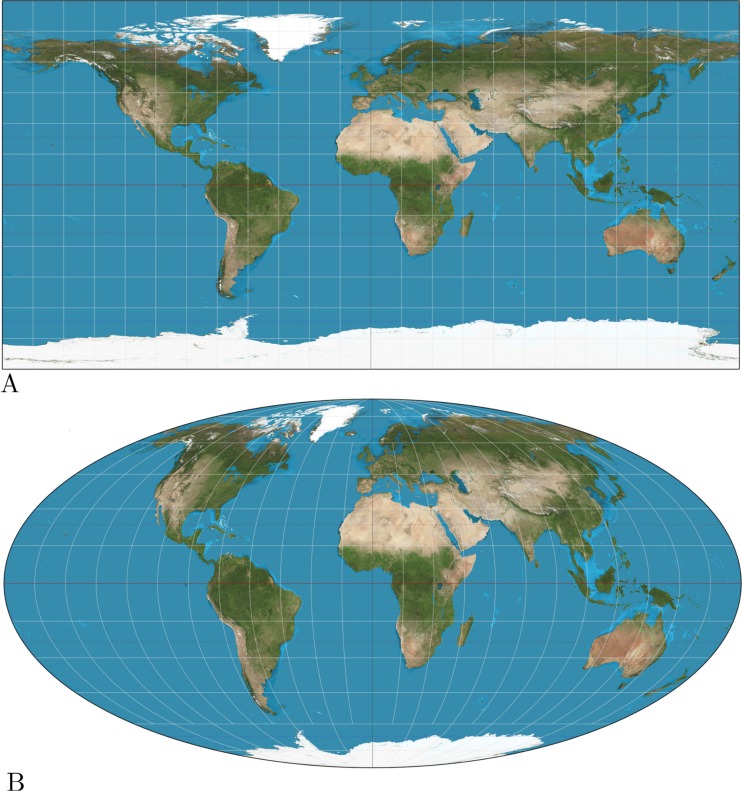
(A) Equirectangular projection, (B) Mollweide projection. The equirectangular projection shows large distortions in distance when moving away from the poles, while the Mollweide projection suffers from distortions in shape.

The assumption underlying these latter methods is that taxa migrate through a random walk over a plane. This may be appropriate for smaller areas, but when samples are taken from a large area of the planet, it may be necessary to map part of the planet onto a plane. Figure 1 shows two such projections: the popular equirectangular projection, which shows large distortions in areas especially around the poles and the Mollweide projection which ensures equal areas, but distorts the relative positions. Though some projections preserve some metric properties, unfortunately, there is no projection that maintains distances between all pairs of points (see Snyder (1987) for a review of cartographic projections).

In this paper, we consider random walks over a sphere, instead of over a plane. This is equivalent to assuming a heterogeneous diffusion process over our planet instead of having it being distorted by cartographic projections. The benefit is that we do not need to worry about distortions in the projection. Also, for smaller areas it behaves equivalent to a model assuming diffusion over a plane, so it can be applied on any scale. Perhaps the most closely related model is described in Drummond (2002), which uses a less accurate approximation for spherical diffusion and does not work out an efficient inference scheme.

**Figure 2:**
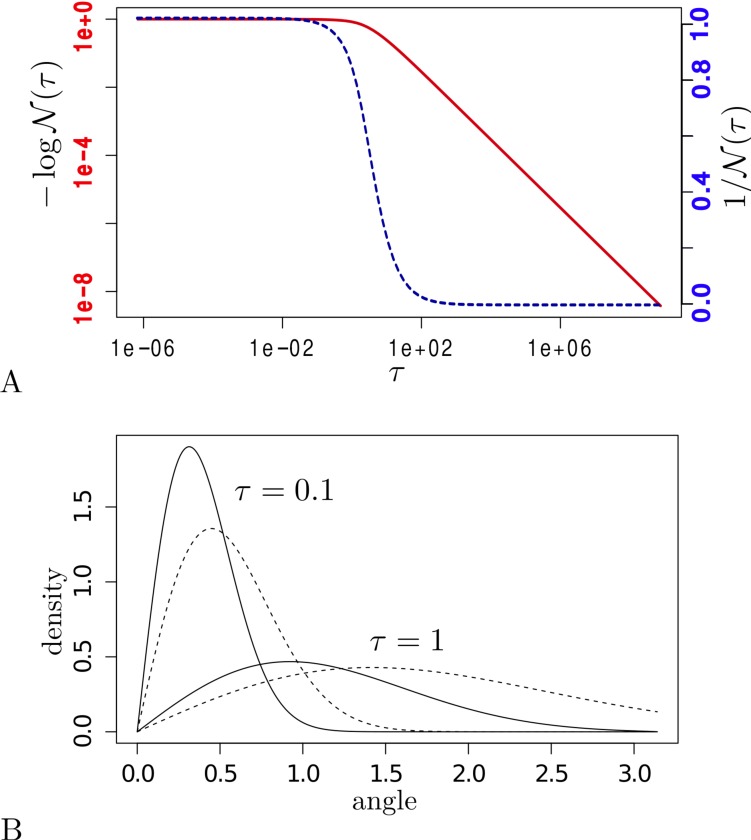
(A) $1{}\mathcal{N}(\mathrm{&tau;})$ (*y*-axis) for different values of *τ* = *b*∕*t* (*x*-axis) on a log–log scale (left axis, solid line) and normal-log scale (right axis, dashed line). (B) density functions (*y*-axis) for spherical diffusion (solid lines) and planar diffusion (dashed lines) with *τ* = 0.1 and *τ* = 1.0. The *x*-axis is the angle for spherical diffusion and distance to start point for planar diffusion.

## Methods

In this section, we explain the details of the spherical diffusion model and how to apply it to phylogeography. First, we detail the model, which follows the tradition of treating the geography as just another trait on each of the leaves in the tree. In this model, the geography is independent of any sequence information for the leaves conditioned on the tree. We follow this with implementation details for using the model efficiently with MCMC, and develop an approximate likelihood that can be calculated efficiently and relies on setting the internal locations to their (weighted) mean values.

We assume homogeneous diffusion over a sphere, governed by a single parameter, the precision *b* of the diffusion process. For such diffusion, the probability density of making a move over angle *α* at time *t* is closely approximated by Ghosh, Samuel & Sinha (2012):

(1) \begin{eqnarray*}p(\alpha {|}\tau )= \frac{\mathcal{N}(\tau )}{\tau } \sqrt{\sin \nolimits (\alpha )\alpha }\hspace*{1em}{e}^{- \frac{{\alpha }^{2}}{2\tau } }\end{eqnarray*}

where $\mathcal{N}(\mathrm{&tau;})$ is a normalising condition such that $1/\mathcal{N}(\tau )=\int \nolimits _{0}^{\pi }p(\alpha {|}\tau )\mathrm{d}\alpha =1$ and $\tau = \frac{t}{b} $. Figure 2 shows the shape of $\mathcal{N}(\mathrm{&tau;})$ for different values of *τ* calculated using numeric integration in R (Ihaka & Gentleman, 1996). For small values of *τ* (<10^−6^) we found that $\mathcal{N}(\mathrm{&tau;})$ is approximately 1 (>0.9999999), while for large values of *τ* (>10^6^) $\mathcal{N}(\mathrm{&tau;})$ approaches $ \frac{2.88266}{\tau } $ within a relative error bounded by 10^−6^. In between we can pre-calculate the values of *τ* in the range 2^*a*∕2^ for *a* ∈ { − 40, 60} and get reasonably close values by interpolating between these samples, since the function of $\mathcal{N}(\mathrm{&tau;})$ is very smooth in *τ*.

Figure 2 shows the difference between a density function with precision 1 and at time 1 for both planar diffusion and spherical diffusion. Spherical diffusion peaks a bit earlier, and planar diffusion has a longer tail. The intuition behind this is that there is less space on a sphere when moving out 1 unit than on a plane, so a slightly earlier peak is expected. Also, for a long distances on a sphere on arrives on the other side at a smaller angle, once the sphere is traversed to the other side.

We assume that the tree is binary in the remainder of the paper, though this assumption can be relaxed, but at the cost of increased complexity, in particular of the mathematical details in Appendix S1. Let *T* be a binary tree over a set of *n* taxa *x*_1_, …, *x*_*n*_. Internal nodes *x*_*n*+1_, …, *x*_2*n*−1_ of the tree are numbered *n* + 1, …, 2*n* − 1. By convention, the highest numbered node (2*n* − 1) is the root and we use *π*_*i*_ to denote the index of the parent of node *i*. With each node *x*_*i*_ is associated a location ℓ_*i*_ = (*ϕ*_*i*_, *λ*_*i*_) with latitude *ϕ*_*i*_ and longitude *λ*_*i*_. Then the likelihood of observing a tree *T* given the set of tip locations ℓ_1…*n*_, precision *b* and other parameters *θ* (like branch length, etc.) is

\begin{eqnarray*}p({\ell }_{1\ldots n}{|}T,D)=\int \nolimits _{{\ell }_{n+1}}\ldots \int \nolimits _{{\ell }_{2n-1}}\prod _{i=1\ldots 2n-2}f({x}_{i}={\ell }_{i}{|}{x}_{{\pi }_{i}}={\ell }_{{\pi }_{i}},\theta ,D)f({\ell }_{2n-1}{|}\theta ,D)\mathrm{d}{\ell }_{n+1}\ldots \mathrm{d}{\ell }_{2n-1} \end{eqnarray*}

where the first density *f*(*x*_*i*_ = ℓ_*i*_|*x*_*π*_*i*__ = ℓ_*π*_*i*__, *θ*, *b*) represents the random walk from parent node *x*_*π*_*i*__ to node *x*_*i*_ and the last density represents the root location prior. Since this integral is intractable, we approximate it by MCMC using the following density:

(2) \begin{eqnarray*}p({\ell }_{1\ldots 2n-1}{|}T,D)=l.367\biggl (\prod _{i=1\ldots 2n-2}f({x}_{i}={\ell }_{i}{|}{x}_{{\pi }_{i}}={\ell }_{{\pi }_{i}},\theta ,D)l.369\biggr )f({\ell }_{2n-1}{|}\theta ,b)\end{eqnarray*}

and augment the parameter space with node locations ℓ_*n*+1_…ℓ_2*n*−1_ of the internal nodes in the tree. Here *f*(*x*_*i*_ = ℓ_*i*_|*x*_*π*_*i*__ = ℓ_*π*_*i*__, *θ*, *b*) = *p*(*α*_*i*_|*b*, *t*_*i*_) where *p*(*α*_*i*_|*b*, *t*_*i*_) is the spherical density of Eq. (1) and *α*_*i*_ the angle between location of *x*_*i*_ and its parent, and *t*_*i*_ the ‘length’ of the branch. Note that since we are assuming homogeneous diffusion, the probability density of ending in *x*_*i*_ when starting at its parent is fully determined by the distance between *x*_*i*_ and its parent and otherwise independent of the actual positions of *x*_*i*_ or its parent. The distance between these locations is represented by the angle *α*_*i*_ between the two points on a sphere. This length is equal to the clock rate used for the branch times the length of the branch in the tree. For instance for a strict clock with rate *r*, the length is simply equal to the branch length times *r*. More relaxed models like the uncorrelated relaxed clock (Drummond et al., 2006), have individual rates that can differ for each branch.

For the root density *p*_*root*_(.) = *f*(ℓ_2*n*−1_|*θ*, *D*), we usually take the uniform prior, indicating we have no preference where the root locations is placed. This simplifies Eq. (2) in that the first term becomes a constant, which can be ignored during MCMC sampling, and we can write it as

(3) \begin{eqnarray*}p({\ell }_{1\ldots n}{|}T,b)\propto \prod _{i=1\ldots 2n-2}f({\alpha }_{i}{|}{t}_{i},b).\end{eqnarray*}

Using more complex priors can have consequence for inference, as outlined in the refinements section. To determine the angle between two locations (*ϕ*_1_, *λ*_1_) and (*ϕ*_2_, *λ*_2_) requires a bit of basic geometry:

\begin{eqnarray*}\alpha =\arccos \nolimits l.416\bigl (\sin \nolimits ({\phi }_{1})\sin \nolimits ({\phi }_{2})+\cos \nolimits ({\lambda }_{1})\cos \nolimits ({\lambda }_{2})\cos \nolimits ({\phi }_{2}-{\phi }_{1})l.418\bigr ). \end{eqnarray*}

### Implementation

The simplest, and in many ways most flexible, approach is by data augmentation, that is, to explicitly maintain all locations as part of the state. Calculation of the likelihood for geography on a tree using Eq. (3) (up to a constant) becomes straightforward. Unfortunately, designing MCMC proposals that efficiently sample the state space is hard, especially since we are not dealing with normal distributions.

### Particle filter approach

The particle filter method (Doucet, De Freitas & Gordon, 2001) is another way of approximating the likelihood without augmenting parameter space with locations. We employ the approach to only estimate the geographical likelihood component of the posterior, but leave estimating the tree and all other parameters to be estimated using MCMC (though in theory, the complete posterior can be approximated that way). So, for every sample in the MCMC chain, the likelihood is calculated based on the tree as informed by other data (e.g., a sequence alignment). As a result, the MCMC has a much lower chance of getting stuck in local maxima and less care is required in designing efficient proposals for dealing with the geography.

To calculate the likelihood, we use a number of ‘particles’ and each particle represents the locations of each of the nodes in the tree. Locations of internal nodes are initialised by setting them to the mean locations of their children in a post-order traversal of the tree, which results in a sufficiently good fit of locations to the tree to guarantee quick convergence. Next, particles are perturbed in pre-order traversal as follows: for a location of node *x*_*i*_, randomly *k* locations are sampled in the vicinity of the current location. Out of the *k* locations, one location is sampled proportional to the partial fit of the location. This partial fit is simply the contribution the location provides to the density (Eq. (3)) consisting of

\begin{eqnarray*}p({\alpha }_{i}{|}b,{t}_{i})p({\alpha }_{\mathrm{left}(i)}{|}b,{t}_{\mathrm{left}(i)})p({\alpha }_{\mathrm{right}(i)}{|}b,{t}_{\mathrm{right}(i)}) \end{eqnarray*}

where *x*_left(*i*)_ is the left child of *x*_*i*_ and *x*_right(*i*)_ its right child. For the root node *p*(*α*_*i*_|*b*, *t*_*i*_) is assumed to be constant.

After all particles are perturbed, an equally sized set of particles is sampled (with replacement) from the current set, with probability proportional to the density as defined by Eq. (3). This process quickly converges to a stable likelihood. Furthermore, it does not easily get stuck in local maxima. Unfortunately, in the context of the MCMC algorithm where the likelihood needs to be recalculated many times, this process is still rather slow. Therefore, we explore an approximation based on assigning mean locations to each of the internal nodes.

### Mean location approximation

Suppose we want to set all internal nodes such that they are in the (branch length weighted) middle of its parent and children, and likewise set the root location as the middle of its children. In other words, we would like to calculate the mean location for each internal node defined as

(4) \begin{eqnarray*}{\phi }_{i}={l}_{i}{\phi }_{L(i)}+{r}_{i}{\phi }_{R(i)}+{p}_{i}{\phi }_{P(i)}\end{eqnarray*}

(5) \begin{eqnarray*}{\lambda }_{i}={l}_{i}{\lambda }_{L(i)}+{r}_{i}{\lambda }_{R(i)}+{p}_{i}{\lambda }_{P(i)}\end{eqnarray*}

and at the root node as

(6) \begin{eqnarray*}{\phi }_{i}={l}_{i}{\phi }_{L(i)}+{r}_{i}{\phi }_{R(i)}\end{eqnarray*}

(7) \begin{eqnarray*}{\lambda }_{i}={l}_{i}{\lambda }_{L(i)}+{r}_{i}{\lambda }_{R(i)}\end{eqnarray*}

where *L*(*i*), *R*(*i*) and *P*(*i*) the index of left and right child and parent of *x*_*i*_ respectively and *l*_*i*_, *r*_*i*_ and *p*_*i*_ are positive weights associated with first and second child and parent respectively that add to unity (*l*_*i*_ + *r*_*i*_ + *p*_*i*_ = 1). We set these weights proportional to the reciprocal of the root of branch lengths.

So, we have a set of *n* − 1 linear equations in *n* − 1 unknown values for latitude, and the same for longitude. We can solve these with standard methods for solving linear equations such as Gaussian elimination in *O*(*n*^3^), but the structure of the tree allows us to solve this problem more in linear time (*O*(*n*)) as follows.

First, do a post order traversal where for each node, we send a message (*m*_*i*_, *ρ*_*i*_) to the parent where if *x*_*i*_ is a leaf node,

\begin{eqnarray*}{m}_{i}={\phi }_{i} \end{eqnarray*}

\begin{eqnarray*}{\rho }_{i}=0 \end{eqnarray*}

and if *x*_*i*_ is not a leaf node,

\begin{eqnarray*}{m}_{i}= \frac{{l}_{i}{m}_{L(i)}+{r}_{i}{m}_{R(i)}}{1-{l}_{i}{\rho }_{L(i)}-{r}_{i}{\rho }_{R(i)}} \end{eqnarray*}

\begin{eqnarray*}{\rho }_{i}= \frac{1}{1-{l}_{i}{\rho }_{L(i)}-{r}_{i}{\rho }_{R(i)}} . \end{eqnarray*}

At the root, we have

(8) \begin{eqnarray*}{\phi }_{i}= \frac{{l}_{i}{m}_{L(i)}+{r}_{i}{m}_{R(i)}}{1-{l}_{i}{\rho }_{L(i)}-{r}_{i}{\rho }_{R(i)}} .\end{eqnarray*}

Next, we do a pre-order traversal from the root, sending down the latitude, and calculate for all internal nodes (but not for leaf nodes)

(9) \begin{eqnarray*}{\phi }_{i}={m}_{i}+{\rho }_{i}{\phi }_{P(i)}.\end{eqnarray*}

Theorem 1 *Using Eqs. (8) and (9), *ϕ*_*i*_ is the mean location as defined in Eqs. (4) and (6) and is calculated in *O*(*n*)*.

**Figure 3:**
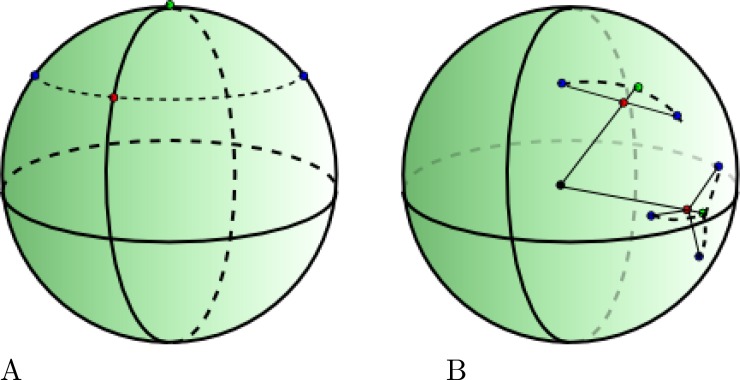
(A) The mean of two blue points by taking the mean latitude/longitude is the red point, while the green point is closer. (B) Taking the mean location of 2 and 3 points on a sphere which leads to quite different and more intuitive mean locations.

See Appendix S1 for a proof. Replacing *ϕ*_*i*_ with *λ*_*i*_ in the above gives the longitudes instead of latitudes. Especially close to the poles, taking the average point between to pairs of latitude/longitude pairs can be a point that is far from the point with the shortest distance to these points. For example the points (45, 0) and (45, 180) (blue points in Fig. 3) on opposite sides of the pole have a mean of (45, 90) (red point)–a point at the same latitude–while the pole (90, 0) (green point) has a shorter distance to these two points.

Instead of using latitude/longitude to represent location, we can use Cartesian coordinates (*x*, *y*, *z*) on a sphere. We can convert (*ϕ*, *λ*) locations to Cartesian using

\begin{eqnarray*}(x,y,z)=(\sin \nolimits (\theta )\cos \nolimits (\phi ),\sin \nolimits (\theta )\sin \nolimits (\phi ),\cos \nolimits (\theta )) \end{eqnarray*}

where *θ* = *λ* × *π*∕180 and *ϕ* = (90 − *ϕ*)*π*∕180. To convert a Cartesian point back to latitude/longitude, we use

\begin{eqnarray*}(\phi ,\lambda )=(\arccos \nolimits (-z)180/\pi -90,\arctan \nolimits 2(y,x)180/\pi ) \end{eqnarray*}

where arctan2 is the arctangent function with two arguments. However, the mean of two points on a sphere is not necessarily on a sphere. But if we take the mean of two points and project it onto a sphere by taking the intersection of the sphere with a line through the origin and the mean point, we get the point that has shortest distance to both points. The algorithm calculating the mean latitude and longitude as defined in Eqs. (4) and (6) readily generalises to the mean of locations on a sphere in three dimensions, so that is what we use from here onwards.

**Figure 4:**
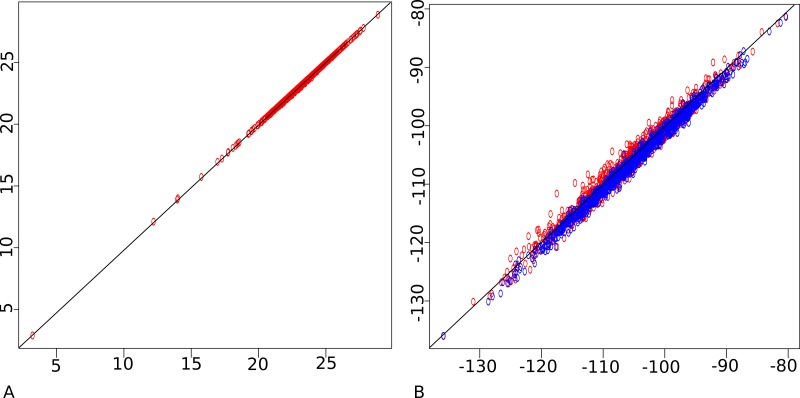
Particle filter (horizontal) vs mean approximation (vertical) on a log of log-likelihood scale. (A) sampled from prior, (B) sampled from posterior (in blue). Also particle filter vs particle filter (in red) showing stochasticity of particle filter. This shows the approximation agrees with the much more computational intensive particle filter.

Figure 4 shows the likelihood calculated through the particle filter approximation and the mean location approximation outlined above. When sampling from the prior, the correlation is almost perfect, while when sampling from the posterior, the mean approximation shows a slight bias. Also shown in Fig. 4 is how well the particle filter correlates with itself. Due to the stochastic nature of the algorithm, this correlation is not 100%, and only slightly better than the correlation with the mean approximation approach.

In summary, we can use the mean approximation to determine locations of internal nodes using a fast *O*(*n*) algorithm and use Eq. (3) with these locations to approximate the likelihood. The only geography specific parameter to be sampled by the MCMC is the precision parameter for the diffusion process. In order to visualise the inferred geography, we need to create a log of locations during the MCMC run. However, logging the mean approximations would result in biased estimates of the uncertainty in locations. To prevent this, a particle filter is run for those samples that are logged and one of the particles containing all internal locations is used as representative sample for a particular state of the MCMC. Since the particle filter only needs to be run when a state is logged, this is sufficiently computationally efficient to be practical.

**Figure 5:**
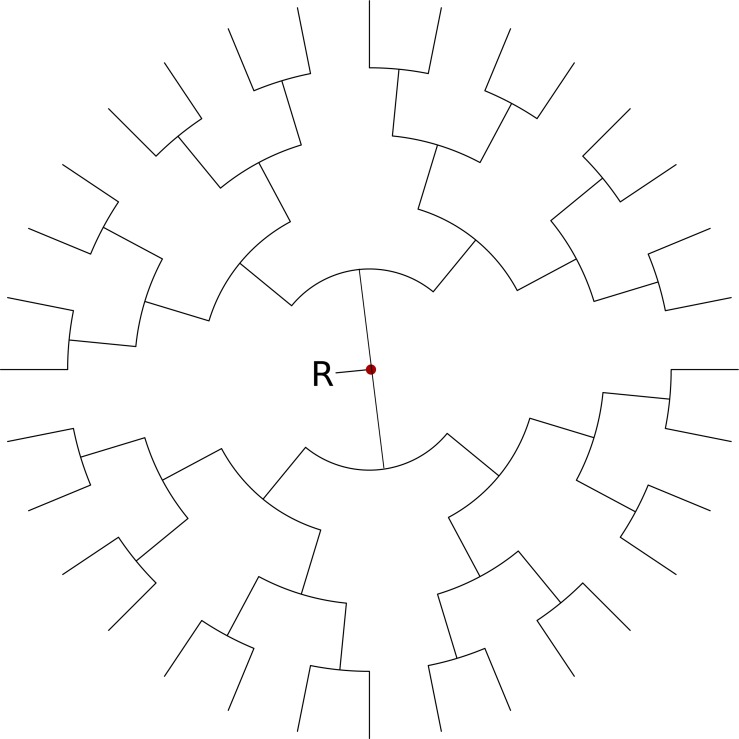
Balanced tree for simulation study with tip locations in latitude, longitude pairs evenly distributed over a circle centred on (0, 0) and radius of 5°. In the simulation study, we rotate these points over different angles ranging from 0 to 90°.

## Results

To compare the planar and spherical diffusion models, we ran an MCMC analysis with both models using a fixed tree with 32 taxa that are positioned on a sphere around the location (0, 0) (see Fig. 5). The same coordinates were then placed on the sphere, the sphere rotated towards the south pole in steps of 10°up to 90°and resulting latitude and longitude positions used for tips. So, the great circle distance between the tips does not change when rotated. An equirectangular (plate carrée) projection was used for the planar diffusion model, so a point represented by (*ϕ*, *λ*) is mapped onto the point (*x*, *y*) on a map with *x* = *ϕ* and *y* = *λ*. One would expect when inferring the root locations (R in Fig. 5) that rotating it back with the same angle would result in inferring the same root location as for the unrotated problem.

Table 1 shows the difference in original and rotated roots when the estimated root is rotated back. The planar diffusion shows a considerable difference in the estimate of the latitude, while it does not for the spherical diffusion estimate. The spherical diffusion model shows smaller standard deviation, possibly due to a different parameterisation than the planar model. Both models contain the original root location in the 95% highest probability density (HPD) interval, except when the tip locations are 90°rotated, then the planar diffusion model does not contain the root location any more. The spherical diffusion model has a considerably lower bias than the planar model. Longitude estimates for both models are very close to the expected value for both models. Note that 95% HPD interval sizes are comparable for both diffusion models at lower amount of rotations, but are erratic at higher amount of rotation for planar diffusion.

**Table 1:** Difference in root location between unrotated and rotated cases for planar and spherical diffusion models. The numbers in brackets represent the 95% HPD for the difference of expected and estimated angle.

Angle	Diffusion on plane				Diffusion on sphere			
	Δ latitude		Δ longitude		Δ latitude		Δ longitude	
0	0.00	(−2.19, 2.26)	0.01	(−1.94, 1.86)	−0.03	(−2.11, 1.82)	0.01	(−1.83, 1.99)
10	0.02	(−2.22, 2.22)	0.00	(−1.87, 1.89)	−0.05	(−2.84, 2.52)	0.03	(−2.95, 2.42)
20	0.07	(−2.21, 2.21)	0.00	(−1.91, 1.87)	−0.02	(−2.20, 2.69)	0.01	(−2.45, 2.70)
30	0.11	(−2.04, 2.42)	0.00	(−1.86, 1.88)	−0.09	(−2.42, 2.52)	0.10	(−2.42, 2.42)
40	0.16	(−2.16, 2.32)	−0.02	(−1.97, 1.84)	−0.17	(−2.81, 2.18)	−0.03	(−2.43, 2.23)
50	0.18	(−2.06, 2.38)	0.00	(−1.86, 1.88)	−0.04	(−2.45, 2.10)	−0.09	(−2.07, 2.31)
60	0.25	(−2.01, 2.50)	0.00	(−1.84, 1.94)	−0.20	(−2.51, 1.68)	0.00	(−2.11, 1.82)
70	0.37	(−1.92, 2.54)	0.01	(−1.91, 1.90)	−0.18	(−2.01, 1.79)	−0.01	(−1.72, 1.79)
80	0.68	(−1.56, 2.92)	−0.03	(−1.99, 1.93)	−0.22	(−2.03, 1.65)	0.06	(−1.35, 1.42)
90	4.54	(3.25, 5.02)	0.02	(−3.96, 3.56)	−0.01	(−1.44, 1.36)	0.00	(−1.08, 1.01)

**Figure 6:**
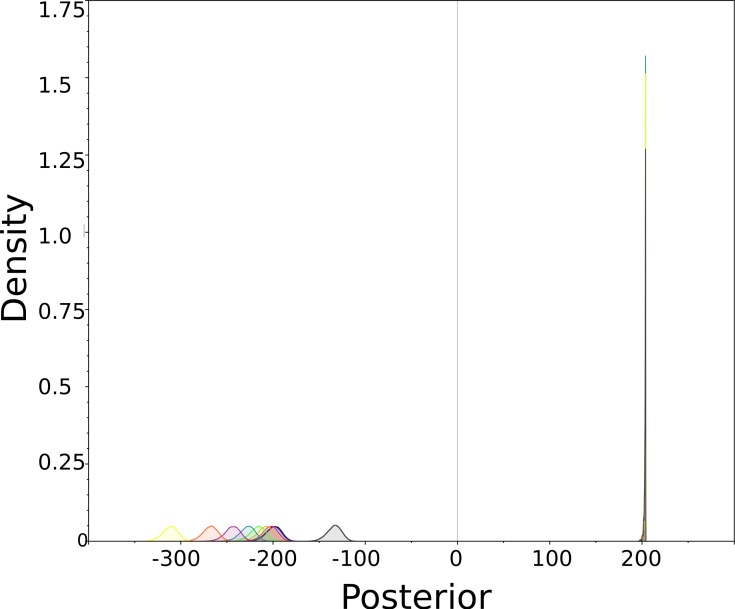
Posterior (=prior × likelihood) densities for planar (left side) and spherical (right side) for simulation study. These are posterior distributions of the marginal likelihood of the entire model smoothed using KDE. Colours indicate individual analyses for different angles as listed in Table 1. Since the positions of taxa relative to each other did not change, it would be desirable for the density to remain the same, as they do for the spherical diffusion model

Furthermore, the planar diffusion model has different likelihoods and posteriors, while the spherical diffusion model’s posterior is invariant under rotation of the taxa. This is illustrated by Fig. 6 that shows posteriors for all rotated instances. The ones for planar diffusion are clearly distinguishable while the ones for spherical diffusion are too similar to be separated.

To get an impression of the capability of the spherical model, 129 HBV full genome sequences were taken from Genbank (see Appendix S1 for accession numbers, sample dates used and country of origin) and clustalx (Larkin et al., 2007) was used to align the sequences. Samples come from Cambodia, China, DR Congo, France, Germany, Ghana, Greece, India, Indonesia, Italy, Japan, Korea, Myanmar, Namibia, Philippines, Russia, Thailand, Turkey and Vietnam. At the initial analysis, tips were not sampled but just taken from a centroid of the country. In this case, the Russian samples clearly reside in different parts of the tree and as a consequence when visualising the tree through space, branches from both Europe and East Asia converged into the point representing Russian samples. Since such a scenario is unlikely, and it is not possible to get more accurate sample location information, tips were sampled from the regions defined by the country of origin as listed in Appendix S1. Border data was obtained from http://thematicmapping.org/downloads/world_borders.php which is available under the Creative Commons license and converted to KML files at http://www.mapsdata.co.uk/online-file-converter/.

Many attempts at estimating the rate for HBV have been made (see, e.g., Bouckaert, Alvarado-Mora & Rebello Pinho, 2013; Paraskevis et al., 2013; Harrison et al., 2011) but most estimates are inconsistent. In order to concentrate on geography, the clock rate was fixed at 2.0E−5 substitutions/site/year, but results can be scaled to what the reader deems more appropriate.

We used BEAST 2 (Bouckaert et al., 2014) to perform the analysis with the generalised time reversible (GTR) substitution model with gamma rate heterogeneity with 4 categories and invariant sites. The uncorrelated relaxed clock with log normal distribution (Drummond et al., 2006) was used and shows a coefficient of variation with a mean of 0.476 and 95% HPD interval of (0.3721, 0.5715), which means a strict clock can be ruled out. A coalescent with constant population size was used as tree prior. The spherical model was run with a strict clock and a relaxed log normal clock to see whether the strict clock could be rejected. The Akaike information criterion (AIC) through MCMC (AICM) values (Baele et al., 2012) of the strict clock was 69215 ± 0.93 while that of the relaxed clock was 69086 ± 2.063 thus favouring the relaxed clock with a difference of over 128.

**Figure 7:**
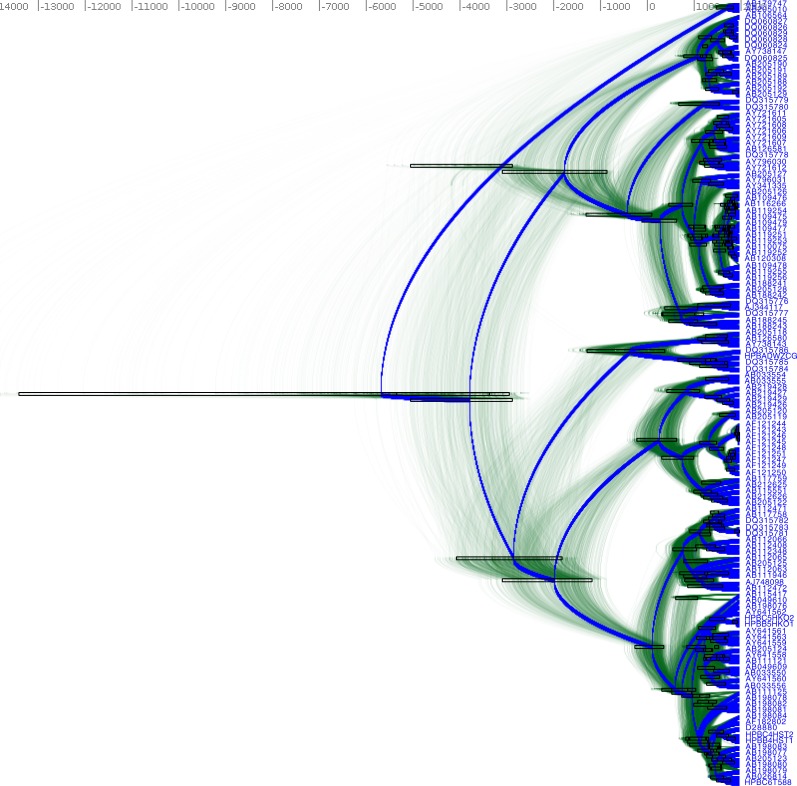
DensiTree of Hepatitis B in Eurasia and Africa. Figure 8 shows the accompanying geographical reconstruction.

**Figure 8:**
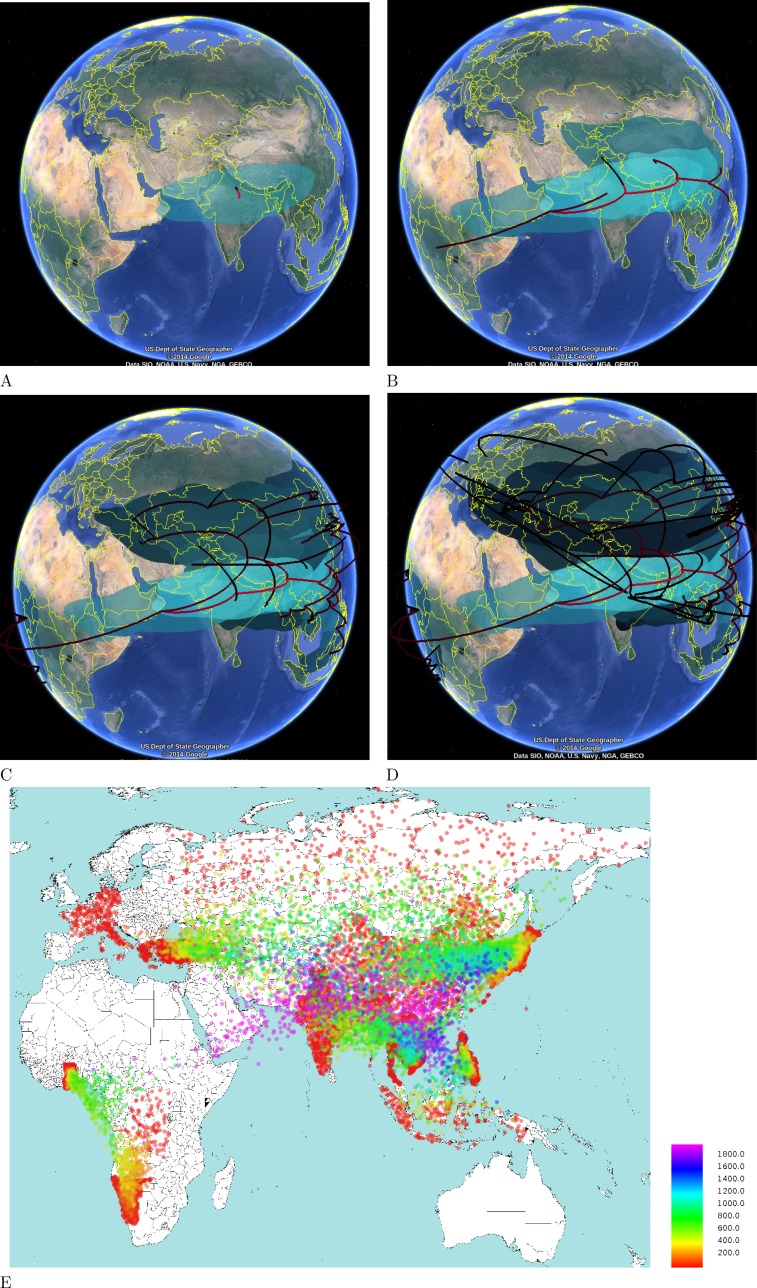
Hepatitis B in Eurasia and Africa through time. The four time slices are at (A) 4000 BCE, (B) 2000 BCE, (C) 1000 CE and (D) 2000 CE. Coloured areas indicate posterior distributions smoothed using two dimensional kernel density estimation. (E) An alternative heat map representation is shown at the lower half, where each dot is an internal node in the posterior set of trees coloured according to age. This highlighs that most of the migration occurred in the last 2000 years. Ages of dots are as indicated in the legend.

Figure 7 shows the DensiTree (Bouckaert & Heled, 2014) of the HBV analysis, which demonstrates it is fairly well resolved, with many clades having 100% posterior support. Fig. 8 shows the tree mapped onto the earth after processing with SPREAD (Bielejec et al., 2011) and visualised with Google-earth. The root of the tree and thus the associated origin of the virus is placed in northern India about 10,000 year ago. Figure 8 shows the spread at times −4000 CE, −1000 CE, 1000 CE and present. Also shown is a heat map visualising the internal node positions of the trees in tree set representing the posterior. Colours indicate age of the internal node as shown in the legend. It suggests that most of the spread happened relatively recently in the last 2000 years. Most of Africa remains white due to lack of samples in the data set in those areas.

## Discussion

The spherical diffusion model can be used for phylogeographical analyses in situations similar to where the models of Lemey et al. (2010) and Pybus et al. (2012) are used, which are assuming diffusion on a plane. Furthermore, the spherical diffusion model can be used when the area of interest is large, and there is considerable distortion when the area is projected on a plane.

However, it assumes heterogeneous diffusion over a sphere. This means that, unlike the model from Lemey et al. (2010), no distinction is made between possible correlations in the direction of the random walk. Furthermore, the random walk on a sphere assumes a Gaussian distribution with relatively thin tails compared to a Levy jump process on a plane (Pybus et al., 2012). Also, it assumes heterogeneous diffusion, unlike the landscape aware model (Bouckaert et al., 2012).

The spherical diffusion model assumes locations in continuous space, which tends to be more powerful than using discrete locations. However, it also means that phylogeographical approaches based on the structured coalescent (Hudson, 1990) cannot be applied, hence demographic developments are not captured by the geographical process.

Note that Ghosh, Samuel & Sinha (2012) pointed out that the approximation of Eq. (1) is very good for small to medium values of *τ* and showed some deterioration when *τ* becomes large, and deterioration much less than shown in Fig. 2 when using planar diffusion instead of spherical diffusion. So, when performig an analysis, it should be verified that the values of *τ* in use are indeed small enough to warrant using the approximation. A good first indication is when the estimated precision *b* is much higher than the height of the tree, since the average $\tau = \frac{b}{t} $ and the length *t* of any branch cannot exceed the tree height, thus *τ* will be much smaller than 1 on average. For the HBV analysis, we found that the maximum *τ* is 0.8 (0.25–1.6 95% HPD), while the mean value of *τ* (averaged over all branches) converged to much smaler values (0.016 [0.011, 0.023] 95% HPD), which is in the range where the approximation (1) is very good.

The method is implemented in the GEO_SPHERE package in BEAST 2 (Bouckaert et al., 2014; Drummond & Bouckaert, 2015), which is open source and licensed under LGPL. An analysis can be set up using BEAUti, the graphical user interface for BEAST. Since it is a BEAST package, it takes little effort to set up an analysis performing joint tree and geography analysis where the tree is mostly informed by sequence data, like DNA sequences, cognate data for languages, or morphological characters. Multi-species coalescent analyses informing a species tree used for geographical inference is also a possibility. Results can be visualised using Google-earth after processing with SPREAD (Bielejec et al., 2011) or the heat map utility that is part of the GEO_SPHERE package. A tutorial explaining how to use the method and set up an analysis is available from http://beast2.org/tutorials.

### Refinements

The mean approximation outlined above assumes tips have fixed positions and all internal nodes do not. Often the sample location for a tip is not exactly known, but a region with known boundaries, for example a country or province is available. In that case, the tips can be sampled using a uniform prior over the region it is known to be sampled from. A random walk proposal for tips can be used to sample tip locations uniformly from the region allowed by the prior. The mean approximation runs as before, but obviously when a tip is updated, the tip location needs to be fixed at the new location for calculating Eq. (3). Note the MCMC proposal allows sampling from disjoint regions like in Nylinder et al. (2014). It was suggested that using uniform distributions for geographical priors may emphasise peripheral regions more than can be justified, thus influencing the geographical inference (Nylinder et al., 2014). In this framework, it should be straightforward to specify non-uniform priors over tip locations or priors on roots and internal clades.

Suppose a globally uniform prior for the root is not appropriate, but a region is known to which the root can be confined. The mean-approximation will assign a value to the root location without concern for such prior and it may assign a location to the root outside the known region. However, if the root location is represented explicitly as part of the state for the MCMC algorithm, the mean approximation can use that location in its likelihood calculation instead the one represented by Eqs. (6) and (7). Like tip locations, the root location can be sampled. A distribution representing whether the root locations is in the region can be added to the prior.

The same technique can be applied if the region of a clade represented by a set of taxa is known. Such location can be explicitly modelled in the state (through data augmentation) replacing the mean location approximation to provide node locations for the density (3).

## Conclusions

We introduced a new framework to perform Bayesian phylogeographical analyses based on diffusion on a sphere. An approximation of the likelihood that can be calculated efficiently was presented, which can be used in an MCMC framework and is implemented in BEAST 2. The method allows branch rate models in order to relax the strict clock assumption, as well as efficient sampling when prior information in the form of sampling regions for tip, root or monophyletic clade locations is available.

Further investigations include incorporating inhomogeneous random walks to represent more realistic diffusion processes that distinguish different rates among land and water, among forests and deserts, etc. A landscape aware model was presented in Bouckaert et al. (2012), but due to its computationally expensive way of preprocessing the method is not very practical. A more efficient method is required to allow testing whether landscape or other features have an impact on migration patterns.

Sample locations are often only approximately known, for example, by the country of origin. The current framework allows sampling such locations from uniform priors as well as non-uniform priors, though only the former are implemented at the moment. Since uniform priors can put an unrealistic amount of support on peripheral areas (Nylinder et al., 2014) it will be interesting to see the quantitative impact of using non-uniform priors for tip locations and internal nodes.

##  Supplemental Information

10.7717/peerj.2406/supp-1 Appendix S1 Appendices Proof of theorem and list of HBV sequences used in HBV analysis.

## Additional Information and Declarations

### Data Availability

The following information was supplied regarding data availability: Source code for the GEO_SPHERE package is available from https://github.com/BEAST2-Dev/beast-geo.
