# Supplementary material for: Phylogeography by diffusion on a sphere: whole world phylogeography
Source: PeerJ. 2016 Sep 6;4:e2406. doi: 10.7717/peerj.2406 (PMC5018680; doi:10.7717/peerj.2406)
Supplement: Appendix S1 — Proof of theorem and list of HBV sequences used in HBV analysis. [file peerj-04-2406-s001.pdf]

Appendix A: Proof of Theorem

Proof. First, we show that Equation (9) and (8) are valid for any internal node x_i when calculated by post-order traversal. When we reach x_i it can be an internal node or the root node. If x_i is an internal node, we distinguish between the children being leaf nodes or internal nodes. Images for Figure 1 were taken from Wikipedia.

1) $x_{L(i)}$ *and* $x_{R(i)}$ *are leaf nodes.* Then Equation (4) gives, (using $\phi_{L(i)} = m_{L(i)}$, $\phi_{R(i)} = m_{R(i)}$ for child nodes $x_L(i)$ and $x_R(i)$, and the definition of m_i and ρ_i)

$$\begin{aligned}
\phi_i &= l_i\phi_{L(i)} + r_i\phi_{R(i)} + p_i\phi_{P(i)} \\
&= l_i m_{L(i)} + r_i m_{R(i)} + p_i\phi_{P(i)} \\
&= \frac{l_{L(i)}m_{L(i)} + r_{R(i)}m_{R(i)}}{1 - l_{L(i)}0 - r_{R(i)}0} + \frac{p_i}{1 - l_{L(i)}0 - r_{R(i)}0}\phi_{P(i)} \\
&= m_i + \rho_i\phi_{P(i)}.
\end{aligned}$$

2) $x_{L(i)}$ *is a leaf and* $x_{R(i)}$ *an internal node.* Then, $\phi_{R(i)} = m_{R(i)} + \rho_{R(i)}\phi_i$ (by post-order traversal) and $\phi_{L(i)} = m_{L(i)}$. Then Equation (4) gives,

$$\begin{aligned}
\phi_i &= l_i\phi_{L(i)} + r_i\phi_{R(i)} + p_i\phi_{P(i)} \\
&= l_i m_{L(i)} + r_i(m_{R(i)} + \rho_{R(i)}\phi_i) + p_i\phi_{P(i)}
\end{aligned}$$

grouping ϕ_i and divide by $1 - 0 - r_i\rho_{R(i)}$ gives

$$\begin{aligned}
lat_i &= \frac{l_{L(i)}m_{L(i)} + r_{R(i)}m_{R(i)}}{1 - l_{L(i)}0 - r_{R(i)}\rho_{R(i)}} + \frac{p_i}{1 - l_{L(i)}0 - r_{R(i)}\rho_{R(i)}}\phi_{P(i)} \\
&= \frac{l_{L(i)}m_{L(i)} + r_{R(i)}m_{R(i)}}{1 - l_{L(i)}\rho_{L(i)} - r_{R(i)}\rho_{R(i)}} + \frac{p_i}{1 - l_{L(i)}\rho_{L(i)} - r_{R(i)}\rho_{R(i)}}\phi_{P(i)} \\
&= m_i + \rho_i\phi_{P(i)}.
\end{aligned}$$

3) $x_{L(i)}$ *is an internal node and* $x_{R(i)}$ *a leaf node.* By symmetry, case 2 holds.

4) $x_{L(i)}$ *and* $x_{R(i)}$ *are both internal nodes.* Then, $\phi_{L(i)} = m_{L(i)} + \rho_{L(i)}\phi_i$ and $\phi_{R(i)} = m_{R(i)} + \rho_{R(i)}\phi_i$(by post-order traversal). Now Equation (4) gives,

$$\begin{aligned}
\phi_i &= l_i\phi_{L(i)} + r_i\phi_{R(i)} + p_i\phi_{P(i)} \\
&= l_i(m_{L(i)} + \rho_{L(i)}\phi_i) + r_i(m_{R(i)} + \rho_{R(i)}\phi_i) + p_i\phi_{P(i)}
\end{aligned}$$

grouping ϕ_i and divide by $1 - l_i\rho_{L(i)} - r_i\rho_{R(i)}$ gives

$$\begin{aligned}
lat_i &= \frac{l_{L(i)}m_{L(i)} + r_{R(i)}m_{R(i)}}{1 - l_{L(i)}\rho_{L(i)} - r_{R(i)}\rho_{R(i)}} + \frac{p_i}{1 - l_{L(i)}\rho_{L(i)} - r_{R(i)}\rho_{R(i)}}\phi_{P(i)} \\
&= m_i + \rho_i\phi_{P(i)}.
\end{aligned}$$

If x_i is the root Equation (8) follows from a similar argument, but with $\rho_i = 0$.

From equations Equations (9) and (8) together it follows that the mean can be calculated by post-order traversal to provide information for the root location (8) to be calculated, followed by a pre-order traversal to calculate locations of internal nodes using (9).

Complexity: it is easy to see the algorithm is $O(n)$ since it takes one post-order traversal sending $2n - 2$ messages up and one pre-order traversal calculating locations for $n - 1$ internal nodes. Each message and latitude calculation is $O(1)$. $\square$

Appendix B: HBV data

Genbank	year	location	Genbank	year	location	Genbank	year	location
AB026814	1998	Japan	AB198076	2001	China	AJ748098	2002	Vietnam
AB033550	1988	Japan	AB198077	2001	China	AY341335	2003	Greece
AB033554	1985	Indonesia	AB198078	2001	China	AY641558	2003	Korea
AB033555	1984	Indonesia	AB198079	2001	China	AY641559	2003	Korea
AB033556	1985	Japan	AB198080	2001	China	AY641560	2003	Korea
AB049609	1996	Japan	AB198081	2001	China	AY641561	2003	Korea
AB049610	1996	Japan	AB198082	2001	China	AY641562	2003	Korea
AB106564	1999	Ghana	AB198083	2001	China	AY641563	2003	Korea
AB109475	2001	Japan	AB198084	2001	China	AY721605	2004	Turkey
AB109476	1997	Japan	AB205010	1994	Japan	AY721606	2004	Turkey
AB109477	1997	Japan	AB205118	2001	Japan	AY721607	2004	Turkey
AB109478	1998	Japan	AB205119	2000	Japan	AY721608	2004	Turkey
AB109479	1997	Japan	AB205120	2000	Japan	AY721609	2004	Turkey
AB110075	2001	Japan	AB205122	2001	Vietnam	AY721611	2004	Turkey
AB111121	1998	Japan	AB205123	2002	China	AY721612	2004	Turkey
AB111125	2001	Japan	AB205124	2003	Japan	AY738143	1997	Germany
AB111946	1998	Vietnam	AB205125	2001	Vietnam	AY738147	1998	DR Congo
AB112063	1998	Vietnam	AB205126	2001	Japan	AY796030	2004	Turkey
AB112065	1998	Vietnam	AB205127	2000	Russia	AY796031	2004	Turkey
AB112066	1999	Myanmar	AB205128	2000	Russia	D23680	1991	Japan
AB112348	1999	Myanmar	AB205129	1999	Ghana	D23681	1992	Japan
AB112408	1999	Myanmar	AB205188	2000	Ghana	D23682	1984	Japan
AB112471	2002	Thailand	AB205189	2000	Ghana	D23683	1984	Japan
AB112472	2002	Thailand	AB205190	2000	Ghana	D23684	1988	Japan
AB115417	1996	Japan	AB205191	2000	Ghana	D28880	1992	Japan
AB115551	2004	Cambodia	AB205192	2000	Ghana	DQ060824	1983	Namibia
AB116266	1987	Japan	AB212625	2004	Vietnam	DQ060825	1983	Namibia
AB117758	2004	Cambodia	AB212626	2004	Vietnam	DQ060826	1983	Namibia
AB117759	2004	Cambodia	AB219426	2002	Philippines	DQ060827	1983	Namibia
AB119251	2002	Japan	AB219427	2002	Philippines	DQ060828	1983	Namibia
AB119252	2002	Japan	AB219428	2002	Philippine	DQ060829	1983	Namibia
AB119253	2000	Japan	AB219429	2002	Philippines	DQ315776	2000	India
AB119254	2003	Japan	AF121243	1995	Vietnam	DQ315777	2003	India
AB119255	2001	Japan	AF121244	1993	Vietnam	DQ315778	2003	India
AB119256	1997	Japan	AF121245	1992	Vietnam	DQ315779	2003	India
AB120308	1982	Japan	AF121246	1998	Vietnam	DQ315780	2002	India
AB126580	2000	Russia	AF121247	1994	Vietnam	DQ315781	2003	India
AB126581	2000	Russia	AF121248	1994	Vietnam	DQ315782	2004	India
AB179747	2002	Italy	AF121249	1998	Vietnam	DQ315783	2003	India
AB188241	1999	Japan	AF121250	1997	Vietnam	DQ315784	2003	India
AB188242	1999	Japan	AF121251	1997	Vietnam	DQ315785	2003	India
AB188243	1999	Japan	AF182802	1992	China	DQ315786	2005	India
AB188245	2000	Japan	AJ344117	1990	France	M57663	1987	Philippines
